# Supplementary material for: Measuring the Metabolic Evolution of Glioblastoma throughout Tumor Development, Regression, and Recurrence with Hyperpolarized Magnetic Resonance
Source: Cells. 2021 Oct 1;10(10):2621. doi: 10.3390/cells10102621 (PMC8534002; doi:10.3390/cells10102621)
Supplement: Supplementary file 1 [file cells-10-02621-s001.zip › cells-1369349-supplementary.pdf]

Supplemental Data

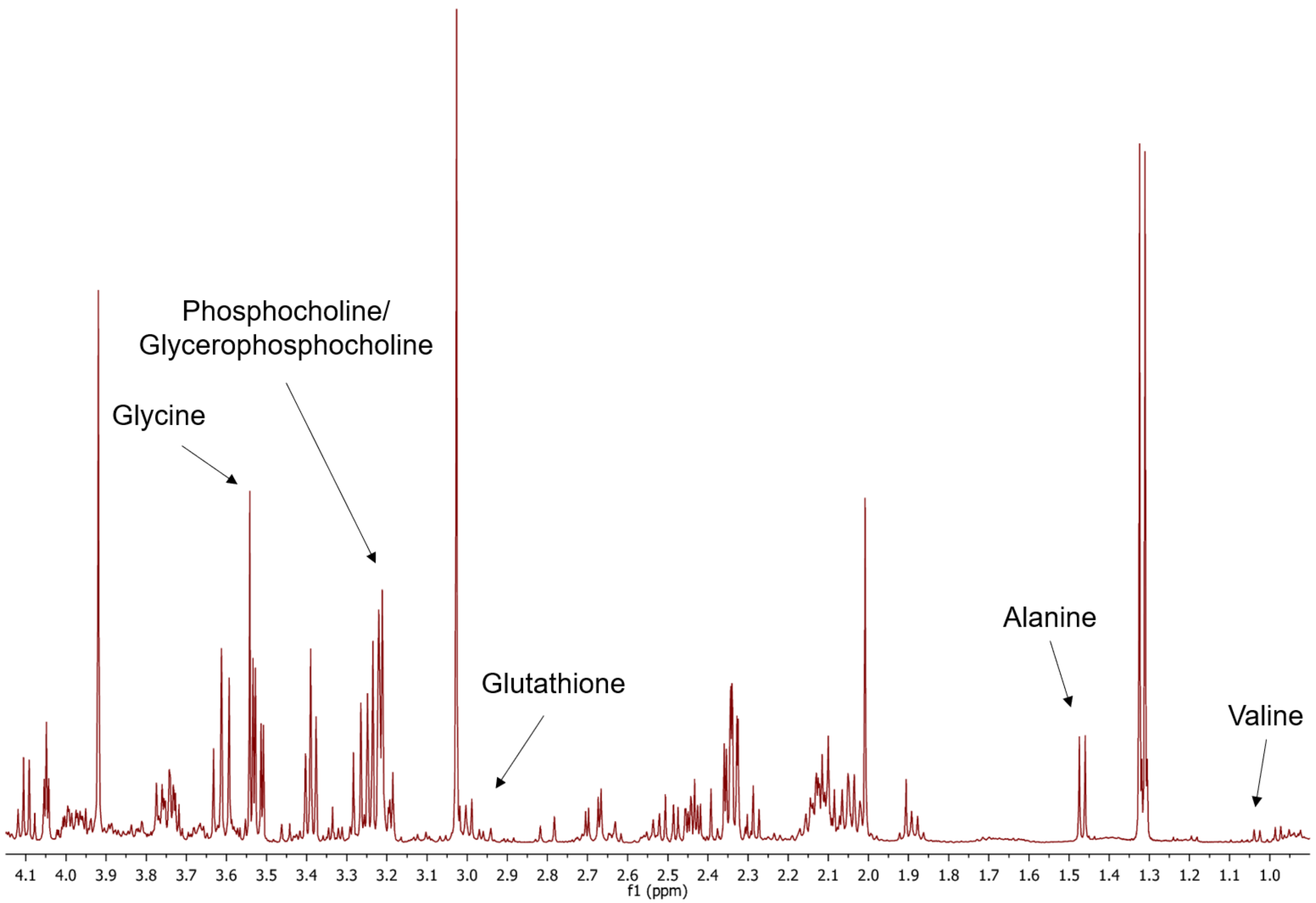


**Figure S1.** Representative NMR spectrum for *ex vivo* metabolomic analysis. Peaks from the metabolites that were significantly altered among controls, untreated/treated are labeled. This particular spectrum was from an untreated tumor on Day 34.
